# Wood heater smoke and mortality in the Australian Capital Territory: a rapid health impact assessment

**DOI:** 10.5694/mja2.52176

**Published:** 2023-11-29

**Authors:** Sotiris Vardoulakis, Fay H Johnston, Nigel Goodman, Geoffrey G Morgan, Dorothy L Robinson

**Affiliations:** ^1^ National Centre for Epidemiology and Population Health Australian National University Canberra ACT; ^2^ Healthy Environments and Lives (HEAL) National Research Network Australian National University Canberra ACT; ^3^ Centre for Safe Air University of Tasmania Hobart TAS; ^4^ Menzies Institute for Medical Research University of Tasmania Hobart TAS; ^5^ Sydney School of Public Health University of Sydney Sydney NSW; ^6^ University Centre for Rural Health University of Sydney Lismore NSW

**Keywords:** Public health, Environmental pollution, Air pollutants, Climate change, Environmental policy

## Abstract

**Objectives:**

To estimate the number of deaths and the cost of deaths attributable to wood heater smoke in the Australian Capital Territory.

**Study design:**

Rapid health impact assessment, based on fine particulate matter (PM_2.5_) data from three outdoor air pollution monitors and published exposure–response functions for natural cause mortality attributed to PM_2.5_ exposure.

**Setting:**

Australian Capital Territory (population, 2021: 454 000), 2016–2018, 2021, and 2022 (2019 and 2020 excluded because of the impact of extreme bushfires on air quality).

**Main outcome measures:**

Proportion of PM_2.5_ exposure attributable to wood heaters; numbers of deaths and associated cost of deaths (based on the value of statistical life: $5.3 million) attributable to wood heater smoke.

**Results:**

Wood heater emissions contributed an estimated 1.16–1.73 μg/m^3^ to the annual mean PM_2.5_ concentration during the three colder years (2017, 2018, 2021), or 17–25% of annual mean exposure, and 0.72 μg/m^3^ (15%) or 0.89 μg/m^3^ (13%) during the two milder years (2016, 2022). Using the most conservative exposure–response function, the estimated annual number of deaths attributable to wood heater smoke was 17–26 during the colder three years and 11–15 deaths during the milder two years. Using the least conservative exposure–response function, an estimated 43–63 deaths per year (colder years) and 26–36 deaths per year (milder years) were attributable to wood heater smoke. The estimated annual equivalent cost of deaths was $57–136 million (most conservative exposure–response function) and $140–333 million (least conservative exposure–response function).

**Conclusions:**

The estimated annual number of deaths in the ACT attributable to wood heater PM_2.5_ pollution is similar to that attributed to the extreme smoke of the 2019–20 Black Summer bushfires. The number of wood heaters should be reduced by banning new installations and phasing out existing units in urban and suburban areas.

**Summary box**

**The known**: Wood heater smoke is a major modifiable source of air pollution in Australian cities and towns that reduces community health and wellbeing.

**The new**: In the Australian Capital Territory, wood heaters contribute an estimated 13–25% of fine particulate matter (PM_2.5_) air pollution; 11–63 premature deaths each year are attributable to PM_2.5_ emitted by wood heaters.

**The implications**: Banning new wood heaters, phasing out existing units in urban and suburban areas, and providing support for a clean domestic energy transition can achieve major health and environmental benefits in Australia.

Domestic wood heaters are the predominant sources of particulate air pollution in the Australian Capital Territory,^1^ as in many other cities and towns in New South Wales,^2^, ^3^ Victoria,^4^ South Australia,^5^ and Tasmania.^6^ Their health impact is substantial: wood heater smoke is responsible for an estimated 269 premature deaths per year in the Sydney Greater Metropolitan Area^7^ and 65 deaths per year in Tasmania.^6^ This effect is larger than for other human sources of air pollution, including road traffic, industry, and power generation.^2^, ^7^ Lower emission alternatives to wood heaters are available.^8^

Wood heater smoke affects neighbourhood air quality and can increase indoor air pollution,^9^ particularly in suburban and regional Australian houses during colder months.^3^ By increasing personal exposure to particulate air pollution, it can exacerbate asthma and other respiratory and cardiovascular conditions.^10^, ^11^, ^12^ A national survey by Asthma Australia in 2020 found that only 28% of respondents and 18% of people with asthma felt able to protect themselves from wood heater smoke when it was present.^13^

The problem of wood heater smoke is more acute in places where air stagnates during winter because of the local topography (eg, in valleys) and temperature inversions (cold polluted air trapped near the ground). Wintertime wood heater pollution is particularly severe in Armidale (NSW) and Tuggeranong (ACT), for example, because of these factors.^1^, ^3^

The use of wood heaters in the ACT is being questioned because of increasing concern about their health and environmental effects.^14^ The 2021 ACT Bushfire Smoke and Air Quality Strategy^1^ recognised wood heaters as a major source of air pollution during colder months, but did not estimate the number of deaths or the associated cost attributable to this source.

We therefore undertook a rapid health impact assessment of the effect of wood heater pollution on mortality in the ACT, based on air quality data from three monitoring stations and published exposure–response functions and population health statistics, to estimate the number of deaths and the associated cost of deaths attributable to wood heater smoke.

## Methods

In our rapid health impact assessment, we estimated the impact of wood heater smoke on mortality in the ACT. We used two exposure–response functions for natural cause mortality derived from a systematic review and meta‐analysis of the effect of a 10 μg/m^3^ increase in long term exposure to PM_2.5_ (fine particulate matter; airborne particles smaller than 2.5 μm diameter):^15^ the first was based on 25 cohort studies (per 10 μg/m^3^ increase: relative risk [RR], 1.08; 95% confidence interval [CI], 1.06–1.09), the second on the five included studies in which the mean PM_2.5_ concentrations were less than 10 μg/m^3^, similar to the annual population exposure level in the ACT (RR, 1.17; 95% CI, 1.12–1.23). We also used an exposure–response function derived from a Queensland study of PM_2.5_ exposure and non‐accidental mortality^16^ (per 10 μg/m^3^ increase in PM_2.5_ exposure: RR, 1.209; 95% CI, 1.145–1.296), where annual mean PM_2.5_ concentrations were between 1.6 and 9.0 μg/m^3^ (Box 1).

Box 1 **Exposure–response functions for natural cause mortality per 10 μg/m^3^ increase in annual PM_2.5_ exposure**

**Table:** 

Source	Includedstudies	Annual PM_2.5_ exposure (μg/m^3^)	Relative risk (95% CI) (per 10 μg/m^3^ increase in PM_2.5_)
Chen and Hoek, 2020 (I)^15^	25	—	1.080 (1.060–1.090)
Chen and Hoek, 2020 (II)^15^		< 10	1.170 (1.120–1.230)
Yu et al, 2020^16^	1	1.6–9.0	1.209 (1.145–1.296)

CI = confidence interval PM_2.5_ = airborne particles of diameter smaller than 2.5 μm.

### Wood heater smoke contribution to PM_2_ _.5_ exposure

To estimate population exposure to wood heater smoke in the ACT (population [2021], 454 000; area, 2358 km^2^),^17^, ^18^ we calculated annual mean PM_2.5_ concentrations measured by the three ACT government air quality monitoring stations:^19^ the Monash station in Tuggeranong Valley (south Canberra), the Florey station in Belconnen (northwest Canberra), and the Civic station in central Canberra. Each station is equipped with high quality PM_2.5_ monitoring instruments (beta‐attenuation monitors), and about 93% of the ACT population reside within 10 km of the three stations. We assumed that the ACT population was exposed to the arithmetic mean of the PM_2.5_ concentrations measured by the three stations.

We assessed PM_2.5_ exposure for the calendar years 2016, 2017, 2018, 2021, and 2022; we excluded 2019 and 2020 because of the influence of the 2019–20 bushfires (“Black Summer”)^20^ on ambient PM_2.5_ levels in the ACT. Based on the annual and diurnal patterns of ambient PM_2.5_ levels in the ACT (Box 2) and responses to a survey on the use of wood heaters,^22^ we defined 1 April – 30 September as the wood heater season. We calculated the mean PM_2.5_ concentrations at the three air quality monitoring sites (overall and by site) for the wood heater season and for the remainder of each calendar year. Three years (2017, 2018, 2021) were relatively cold during the wood heater season (and ambient PM_2.5_ levels higher); two years (2016, 2022) were milder during this period (Supporting Information, figure 1).

Box 2 Mean daily PM_2_ _.5_ concentrations at the three Australian Capital Territory government monitoring stations, 2016–2018, 2021, and 2022*

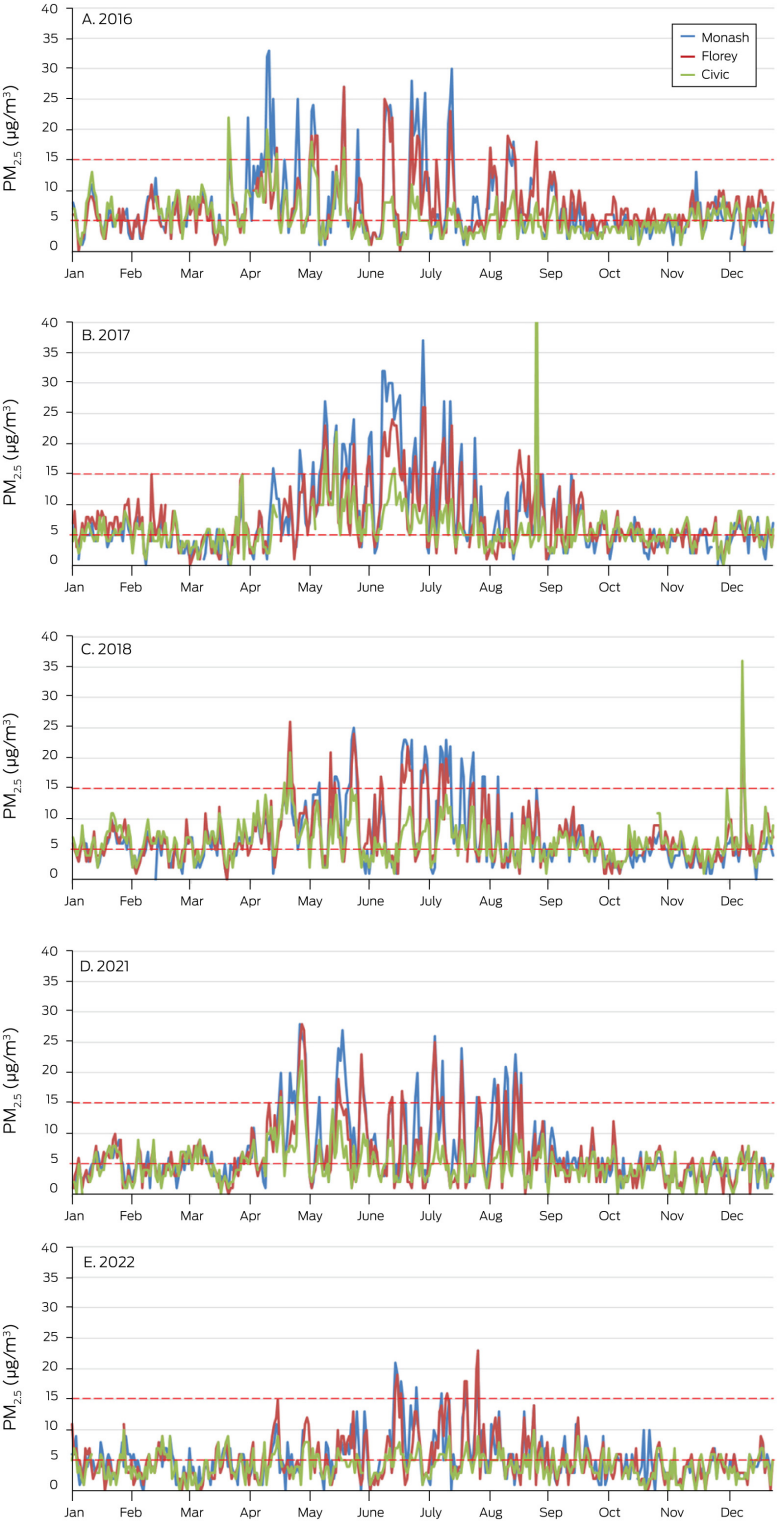

PM_2.5_ = airborne particles of diameter smaller than 2.5 μm.

* Red dashed lines: World Health Organization guidelines for annual mean PM_2.5_ (5 μg/m^3^) and 24‐hour mean concentrations (15 μg/m^3^).^21^ The diurnal patterns are included in the Supporting Information, section 1).

The wood heater contribution to annual PM_2.5_ concentration during the wood heater season was estimated by subtracting the estimated annual mean based on measurements outside the wood heater season from the value based on measurements during the entire year. To account for contributions by other sources of PM_2.5_ during the colder months (eg, prescribed burning in April, May, and September; increased road traffic in winter),^23^, ^24^ we discounted the estimated contribution of wood heating to total PM_2.5_ concentration by 20%. Sensitivity analyses applied 10% or 30% discounts to the wood heater contribution to the PM_2.5_ concentration during the wood heater season.

### Mortality attributable to wood heater smoke

Mortality attributable to wood heater‐related PM_2.5_ exposure was estimated by multiplying the attributable fraction (*AF*) by the mean annual number of deaths in the ACT for the pre‐coronavirus disease 2019 (COVID‐19) pandemic period 2017–2019 (2160 deaths).^25^ To estimate the number of natural adult deaths, we discounted the total number of deaths by 10% to account for those during early childhood (2021: about 0.7% of deaths), and accident‐related and suicide deaths (2021: 9% of deaths).^26^ The attributable fraction (*AF*) was calculated from the relative risk (*RR*):

$$\mathrm{AF}=\left({\mathrm{RR}}_{x}–1\right)/{\mathrm{RR}}_{x}$$

in which *RR*_*x*_ = *(RR*_*10*_ *)* ^*x/10*^ corresponds to a PM_2.5_ incremental increase of *x* μg/m^3^; *RR*_*10*_ is the increase in mortality risk for 10 μg/m^3^ increase in annual PM_2.5_ exposure (ie, the exposure–response function; Supporting Information, section 2).

Based on the value of statistical life of $5.3 million per life in Australia, recommended by the Office of Best Practice Regulation in 2022,^27^ we also calculated the hypothetical cost of deaths attributable to wood heater pollution. The value of statistical life, based on the willingness‐to‐pay approach to reducing mortality risk, has been used in similar studies in Australia^6^, ^28^ and New Zealand;^29^ it does not take the health, age, or life expectancy of individuals or groups into account, nor the costs of treating illness attributable to exposure, including hospitalisation.

### Ethics approval

We did not seek ethics approval, as our estimates were based on data published by other authors,^15^, ^16^ the Australian Bureau of Statistics,^25^ the Australian Institute of Health and Welfare,^26^ the Department of the Prime Minister and Cabinet,^27^ and the Australian Capital Territory government.^19^

## Results

Based on air quality monitoring data from the three ACT monitoring stations, we estimated that wood heater emissions contributed 1.16–1.73 μg/m^3^ to the annual mean PM_2.5_ concentration during the three colder years (2017, 2018, 2021), or 17–25% of annual mean exposure, and 0.72 μg/m^3^ (15%) or 0.89 μg/m^3^ (13%) during the two milder years (2016, 2022) (Box 3).

Box 3 **Mean PM_2.5_ concentrations at Australian Capital Territory government monitoring stations, by year**

**Table:** 

	Mean PM_2.5_ concentration (μg/m^3^)			
Monitor	Wood heater season (Apr–Sept)	Rest of year	Entire year	Wood heating proportion*
Three‐station mean				
2016	7.76	5.54	6.66	0.89 (13%)
2017	9.27	4.95	7.11	1.73 (24%)
2018	8.35	5.44	6.89	1.16 (17%)
2021	7.86	4.16	6.02	1.49 (25%)
2022	5.67	3.88	4.78	0.72 (15%)
Monash				
2016	9.27	5.26	7.27	1.61 (22%)
2017	11.08	4.53	7.84	2.65 (34%)
2018	9.22	4.81	7.01	1.77 (25%)
2021	9.64	4.18	6.95	2.21 (32%)
2022	6.33	4.07	5.17	0.89 (17%)
Florey				
2016	8.41	5.81	7.12	1.05 (15%)
2017	9.67	5.34	7.50	1.73 (23%)
2018	8.81	5.51	7.15	1.31 (18%)
2021	8.07	4.26	6.17	1.53 (25%)
2022	6.16	3.9	5.06	0.93 (18%)
Civic				
2016	5.60	5.56	5.58	0.01 (< 1%)
2017	7.05	4.98	5.99	0.81 (14%)
2018	7.01	6.01	6.52	0.41 (6%)
2021	5.86	4.04	4.95	0.73 (15%)
2022	4.52	3.68	4.10	0.34 (8%)

PM_2.5_ = airborne particles of diameter smaller than 2.5 μm.

* Assumes that 80% of the difference between the annual mean concentration and mean concentration for periods outside wood heater season is attributable to wood heater emissions. Calculations assuming that the contribution is 70% or 90% are included in the Supporting Information, table 1.

Based on these estimates, the estimated annual number of deaths attributable to wood heater PM_2.5_ pollution was 17 to 26 during the colder three years and 11 or 15 during the milder two years using the most conservative exposure–response function, or 43 to 63 (colder years) and 26 or 36 (milder years) using the least conservative exposure–response function (Box 4).

Box 4 Deaths attributable to wood heater emissions in the Australian Capital Territory, by year and exposure–risk function*

**Table:** 

Relative risk source	Deaths (95% confidence interval)				
	2016	2017	2018	2021	2022
Chen and Hoek, 2020 (I)^15^	15 (11–17)	26 (19–29)	17 (13–19)	22 (17–25)	11 (8–12)
Chen and Hoek, 2020 (II)^15^	30 (22–40)	52 (38–68)	35 (25–46)	45 (33–59)	22 (16–29)
Yu et al, 2020^16^	36 (26–49)	63 (45–85)	43 (30–58)	54 (39–74)	26 (19–36)

* Assumes that 80% of the difference between the annual mean concentration and mean concentration for periods outside wood heater season is attributable to wood heater emissions. Calculations assuming that the contribution is 70% or 90% are included in the Supporting Information, tables 2 and 3.

Based on the estimated numbers of deaths, the estimated annual cost of lost health was $92–136 million during the colder three years and $57–78 million during the milder two years using the most conservative exposure–response function, or $225–333 million (colder years) and $140–193 million (milder years) using the least conservative exposure–response function (Box 5).

Box 5 Equivalent cost of deaths attributable to wood heater emissions in the Australian Capital Territory, by year and exposure–risk function*

**Table:** 

Relative risk source	Equivalent cost of deaths, $ million (95% confidence interval)				
	2016	2017	2018	2021	2022
Chen and Hoek, 2020 (I)^15^	78 (59–88)	136 (103–152)	92 (70–103)	117 (89–131)	57 (43–63)
Chen and Hoek, 2020 (II)^15^	159 (115–209)	276 (200–362)	186 (135–245)	238 (172–313)	115 (83–152)
Yu et al, 2020^16^	193 (137–262)	333 (238–452)	225 (161–306)	288 (205–391)	140 (99–190)

* Assumes that 80% of the difference between the annual mean concentration and mean concentration for periods outside wood heater season is attributable to wood heater emissions. Calculations assuming that the contribution is 70% or 90% are included in the Supporting Information, tables 4 and 5.

## Discussion

Wood heaters have long been recognised as major sources of PM_2.5_ pollution in the ACT,^30^ particularly in areas such as the Tuggeranong valley, where air stagnates in winter and wood heaters are particularly numerous. A 2022 government survey found that wood heaters were used for main or supplementary heating in about 11% of occupied private dwellings in the ACT.^14^ A national survey found that people in larger households and with higher household income were more likely to use firewood,^22^ suggesting that affordability is not the main reason for using wood heaters.

We estimated that wood heaters contributed 0.72–1.73 μg/m^3^ to annual mean PM_2.5_ concentrations in the ACT during the five years assessed, or 13–25% of PM_2.5_ pollution from all sources. The estimated contribution of wood heater pollution to population‐weighted PM_2.5_ exposure in Greater Sydney during 2010–11 was 0.49 μg/m^3^ (24% of all human sources);^2^ a different study estimated the contribution in 2013 to be 1.26 μg/m^3^ (42% of all human sources).^7^ A 2012 particle characterisation study in the upper Hunter Valley (NSW) found that 14–30% of the annual PM_2.5_ concentration was attributable to domestic wood heaters.^31^

We estimated the annual equivalent cost of deaths in the ACT attributable to wood heater smoke to be $57–333 million. It was previously estimated that the cost in Greater Sydney (2013) was $2239 million per year;^7^ for Armidale (NSW) it was $32.8 million (95% CI, $27.0–38.5 million) for 2018–19.^3^

As well as affecting local air quality and community health, wood heaters produce carbon dioxide, methane, and black carbon that contribute to climate change. Even were the carbon dioxide emitted by wood heaters fully offset by tree planting (which has not been the case in Australia^32^), wood heaters would contribute more to global warming than efficient reverse cycle air conditioning systems using electricity from renewable sources, which also relieve the need to transport firewood to urban areas, further reducing energy consumption and emissions.^33^

Modern wood heaters typically exceed current emission standards when used under real world conditions,^8^ suggesting that emission control standards are inadequate for protecting health. Further, public education about wood heater use alone does not efficiently improve local air quality.^8^ Reducing the number of wood heaters by not allowing new installations and phasing out existing units is essential for reducing particulate air pollution and its impact on health.^8^, ^14^ Requiring that wood heaters be removed when selling properties could reduce their number significantly.^14^, ^34^ Government rebates and other financial incentives, particularly for low income households, could also encourage people to replace wood heaters with lower emission alternatives, improving domestic energy efficiency and air quality.^14^, ^34^ Australians, particularly those with asthma, generally support regulations for reducing the impact of wood heaters on health; in a 2020 Asthma Australia survey, 77% of respondents agreed that wood heaters should not be allowed in urban areas, and 55% that their use should be phased out.^13^

A more comprehensive network of reference air quality monitors and distributed low and medium cost sensors could characterise population exposure to PM_2.5_ at the neighbourhood level in the ACT, particularly in areas of greater air pollution where people may be particularly vulnerable to wood heater pollution. An up‐to‐date atmospheric emissions inventory and an operational modelling system could test different scenarios and interventions for reducing pollution from domestic wood heaters and other sources.^20^ Low cost community monitoring could assess exposure to PM_2.5_ from wood heaters in residential areas,^35^ better informing regulatory authorities about where protective measures are needed. Finally, an ACT wood heater register would provide more accurate information about the number and age of wood heaters to facilitate estimation of PM_2.5_ emission levels and their impact on health, as well as the evaluation of interventions for reducing them.^8^, ^14^

### Limitations

We made assumptions regarding the duration of the wood heater season in Canberra and the contribution of wood heaters to PM_2.5_ concentrations during the wood heater season. Exposure estimates were based on PM_2.5_ data (without population weighting) from outdoor air quality monitoring sites, and the applicability of exposure–response functions based on studies in other geographic regions^15^, ^16^ is uncertain. We did not take into account the potential differences in PM_2.5_ toxicity from wood heaters and other sources, such as road traffic.^36^ Although exposure to air pollutants emitted by indoor sources can be significant,^37^ we used outdoor PM_2.5_ levels as a surrogate measure of population exposure, as is typical for health impact assessments.^38^ Possible effects on mortality of gaseous pollutants co‐emitted with PM_2.5_ from wood heaters were not taken into account. We report natural cause (ie, non‐accidental) mortality estimates for the overall ACT population rather than cause‐specific mortality or mortality for specific groups of people. Further, as other health‐related consequences of exposure to PM_2.5_ and gases emitted by wood heaters (eg, hospitalisations, general practice visits, deterioration of health symptoms) were not assessed, we probably underestimated the health impact of wood heater pollution in the ACT. Finally, some people, such as those with asthma, are more severely affected by wood heater smoke than the general population.^13^

### Conclusion

The estimated contribution of wood heaters to ambient PM_2.5_ pollution and its impact on public health has caused concern in the ACT.^14^ As there is no safe PM_2.5_ exposure level,^39^ reducing exposure to wood heater smoke, even from levels below current particulate air pollution standards levels, can have substantial benefits for health.^40^ The consequence of current wood heater use in the ACT is 11–63 avoidable deaths, equivalent to $57–333 million in the annual cost of deaths, comparable with the 31 deaths in the ACT attributable to the bushfire smoke during the Black Summer of 2019–20.^41^

Decisive public health action against passive smoking in enclosed public places effectively eliminated harmful exposure to tobacco smoke, a precedent for the potential health benefits of reducing the number of wood heaters in Australia. Cleaner, healthier, and more sustainable and affordable domestic heating options, such as reverse cycle air conditioning powered by energy from renewable sources, can keep Australian homes warm in winter and cool in summer without harming community health and the environment. Making homes more thermally efficient is also important for improving comfort and reducing energy consumption.

More air quality monitoring and modelling is needed to fully assess population exposure and the health impact of air pollution in the ACT, but action should not be delayed. Banning new wood heaters, phasing out existing units in urban and suburban areas, and providing support for a clean domestic energy transition can achieve major health and environmental benefits in Australia.

## Open access

Open access publishing facilitated by Australian National University, as part of the Wiley – Australian National University agreement via the Council of Australian University Librarians.

## Competing interests

No relevant disclosures.

## Supporting information


Supplementary methods and results

## References

[1] Australian Capital Territory Government . Bushfire smoke and air quality strategy 2021–2025. 2021. https://www.act.gov.au/__data/assets/pdf_file/0011/1897859/Bushfire‐smoke‐and‐air‐quality‐strategy‐2021‐2025.pdf (viewed Sept 2023).

[2] Broome RA , Powell J , Cope ME , Morgan GG . The mortality effect of PM_2.5_ sources in the Greater Metropolitan Region of Sydney, Australia. Environ Int 2020; 137: 105429. 32062440 10.1016/j.envint.2019.105429

[3] Robinson DL , Horsley JA , Johnston FH , Morgan GG . The effects on mortality and the associated financial costs of wood heater pollution in a regional Australian city. Med J Aust 2021; 215: 269‐272. https://www.mja.com.au/journal/2021/215/6/effects‐mortality‐and‐associated‐financial‐costs‐wood‐heater‐pollution‐regional 34341997 10.5694/mja2.51199

[4] Legislative Council Environment and Planning Committee (Parliament of Victoria) . Inquiry into the health impacts of air pollution in Victoria (PP no. 294, session 2018–2021). Nov 2021. https://new.parliament.vic.gov.au/4a4c69/contentassets/bfbcb6bd449c4785840cb65d65f28a4c/health‐impacts‐of‐air‐pollution‐victoria.pdf (viewed Sept 2023).

[5] Environment Protection Authority (South Australia) . Smoke from domestic heating. Updated 4 May 2023. https://www.epa.sa.gov.au/environmental_info/air_quality/assistance_and_advice/smoke_from_domestic_heating (viewed Sept 2023).

[6] Borchers‐Arriagada N , Palmer AJ , Bowman DMJS , et al. Health impacts of ambient biomass smoke in Tasmania, Australia. Int J Environ Res Public Health 2020; 17: 3264. 32392847 10.3390/ijerph17093264 PMC7246513

[7] Department of Planning and Environment (New South Wales) . Sydney air quality study. Program report: stage 2. Health impact assessment. 2023. https://www.environment.nsw.gov.au/‐/media/OEH/Corporate‐Site/Documents/Air/sydney‐air‐quality‐study‐program‐report‐stage‐2‐230226.pdf (viewed Sept 2023).

[8] Johnston F , Cubas A , Workman A , et al; Centre for Air Pollution, Energy and Health Research. Reducing the health impacts of wood heaters in Australia. Policy implications [position paper]. Aug 2021. https://8a9fccf2‐785f‐43a0‐af75‐f07582c6bf73.filesusr.com/ugd/d8be6e_a27f05a82f8c47378ffa9dcbacb6cc04.pdf (viewed Sept 2023).

[9] Robinson DL . Air pollution in Australia: review of costs, sources and potential solutions. Health Promot J Austr 2005; 16: 213‐220. 16375037 10.1071/he05213

[10] Bui DS , Burgess JA , Matheson MC , et al. Ambient wood smoke, traffic pollution and adult asthma prevalence and severity. Respirology 2013; 18: 1101‐1107. 23627489 10.1111/resp.12108

[11] Johnston FH , Salimi F , Williamson GJ , et al. Ambient particulate matter and paramedic assessments of acute diabetic, cardiovascular, and respiratory conditions. Epidemiology 2019; 30: 11‐19. 30334919 10.1097/EDE.0000000000000929 PMC6276863

[12] Hime NJ , Marks GB , Cowie CT . A comparison of the health effects of ambient particulate matter air pollution from five emission sources. Int J Environ Res Public Health 2018; 15: 1206. 29890638 10.3390/ijerph15061206 PMC6024892

[13] Asthma Australia . Woodfire heaters and health survey key findings report. 2021. https://asthma.org.au/wp‐content/uploads/2021/03/Asthma‐Australia‐Woodfire‐Heaters‐and‐Health‐Survey‐Report.pdf (viewed Sept 2023).

[14] Office of the Commissioner for Sustainability and the Environment (ACT) . “Burn right tonight” or is there “no safe level of air pollution”? An investigation into wood heater policy in the ACT. Jan 2023. https://envcomm.act.gov.au/wp‐content/uploads/2022/08/OCSE‐Wood‐Heaters‐Report‐A40588031.pdf (viewed Sept 2023).

[15] Chen J , Hoek G . Long‐term exposure to PM and all‐cause and cause‐specific mortality: a systematic review and meta‐analysis. Environ Int 2020; 143: 105974. 32703584 10.1016/j.envint.2020.105974

[16] Yu W , Guo Y , Shi L , Li S . The association between long‐term exposure to low‐level PM_2.5_ and mortality in the state of Queensland, Australia: a modelling study with the difference‐in‐differences approach. PLoS Med 2020; 17: e1003141. 32555635 10.1371/journal.pmed.1003141 PMC7302440

[17] Australian Bureau of Statistics . Snapshot of Australian Capital Territory. High level summary data for Australian Capital Territory in 2021. 28 June 2022. https://www.abs.gov.au/articles/snapshot‐act‐2021 (viewed Sept 2023).

[18] Geoscience Australia . Area of Australia: states and territories. Updated 26 July 2023. https://www.ga.gov.au/scientific‐topics/national‐location‐information/dimensions/area‐of‐australia‐states‐and‐territories (viewed Sept 2023).

[19] Australian Capital Territory Government . Air quality monitoring data. Updated 26 Oct 2023. https://www.data.act.gov.au/Environment/Air‐Quality‐Monitoring‐Data/94a5‐zqnn (viewed Sept 2023).

[20] Vardoulakis S , Jalaludin BB , Morgan GG , et al. Bushfire smoke: urgent need for a national health protection strategy. Med J Aust 2020; 212: 349‐353. https://www.mja.com.au/journal/2020/212/8/bushfire‐smoke‐urgent‐need‐national‐health‐protection‐strategy 32088929 10.5694/mja2.50511 PMC7318141

[21] World Health Organization . WHO global air quality guidelines. Particulate matter (PM_2.5_ and PM_10_), ozone, nitrogen oxide, sulfur dioxide and carbon monoxide. Geneva: World Health Organization, 2021. https://iris.who.int/bitstream/handle/10665/345329/9789240034228‐eng.pdf?sequence=1 (viewed Sept 2023). 34662007

[22] Romanach L , Frederiks E . Understanding the key determinants of residential firewood consumption in Australia: a nationwide household survey. Energies 2021; 14: 6777.

[23] Di Virgilio G , Evans JP , Clarke H , et al. Climate change significantly alters future wildfire mitigation opportunities in southeastern Australia. Geophys Res Lett 2020; 47: e2020GL088893.

[24] de Jesus AL , Thompson H , Knibbs LD , et al. Two decades of trends in urban particulate matter concentrations across Australia. Environ Res 2020; 190: 110021. 32784017 10.1016/j.envres.2020.110021

[25] Australian Bureau of Statistics . Deaths, year of registration, summary data, sex, states, territories and Australia: Australian Capital Territory, 2017–2019. https://explore.data.abs.gov.au/vis?fs[0]=ABS%20Topics%2C0%7CPEOPLE%23PEOPLE%23&fs[1]=ABS%20Topics%2C2%7CPEOPLE%23PEOPLE%23%7CPopulation%23POPULATION%23%7CDeaths%23DEATHS%23&pg=0&fc=ABS%20Topics&df[ds]=ABS_ABS_TOPICS&df[id]=DEATHS_SUMMARY&df[ag]=ABS&df[vs]=1.0.0&pd=2017%2C2019&dq=4..8.A&ly[cl]=TIME_PERIOD (viewed Sept 2023).

[26] Australian Institute of Health and Welfare . Deaths in Australia (Cat. no. PHE 229) [here: tables S2.2 and S5.2, and multiple causes of death summary]. Updated 11 July 2023. https://www.aihw.gov.au/reports/life‐expectancy‐death/deaths‐in‐australia/contents/about (viewed Sept 2023).

[27] Australian Department of the Prime Minister and Cabinet . Best practice regulation guidance note: Value of statistical life. Aug 2022. https://oia.pmc.gov.au/sites/default/files/2022‐09/value‐statistical‐life‐guidance‐note.pdf (viewed Sept 2023).

[28] Johnston FH , Borchers‐Arriagada N , Morgan GG , et al. Unprecedented health costs of smoke‐related PM_2.5_ from the 2019–20 Australian megafires. Nat Sustain 2021; 4: 42‐47.

[29] Kuschel G , Metcalfe J , Sridhar S , et al; Ministry for the Environment; Ministry of Health; Te Manatū Waka Ministry of Transport; Waka Kotahi NZ Transport Agency . Health and air pollution in New Zealand 2016 (HAPINZ 3.0), volume 1. Findings and implications. Mar 2022. https://environment.govt.nz/assets/publications/HAPINZ/HAPINZ‐3.0‐Findings‐and‐implications.pdf (viewed Sept 2023).

[30] Ayers G , Keywood M , Gras J , et al. Chemical and physical properties of Australian fine particles: a pilot study. Final report to Environment Australia from the Division of Atmospheric Research, CSIRO and the Australian Nuclear Science and Technology Organisation. 1999. https://www.cmar.csiro.au/e‐print/open/CSIRO_AFP.pdf (viewed Sept 2023).

[31] Hibberd M , Selleck P , Keywood M , et al. Upper Hunter particle characterization study. Final report [CSIRO Marine and Atmospheric Research]. 17 Sept 2013. https://www.environment.nsw.gov.au/‐/media/OEH/Corporate‐Site/Documents/Air/upper‐hunter‐valley‐particle‐characterization‐study‐final‐report.pdf (viewed Sept 2023).

[32] Robinson DL . Australian wood heaters currently increase global warming and health costs. Atmos Pollut Res 2011; 2: 267‐274.

[33] Australian Department of Climate Change, Energy, the Environment and Water . Wood heaters and woodsmoke. Updated 15 Feb 2023. https://www.dcceew.gov.au/environment/protection/air‐quality/woodheaters‐and‐woodsmoke (viewed Sept 2023).

[34] AECOM Australia . Economic appraisal of wood smoke control measures. Final report (for the NSW Office of Environment and Heritage). 29 June 2011. https://www.epa.nsw.gov.au/~/media/EPA/Corporate%20Site/resources/air/WoodsmokeControlReport.ashx (viewed Sept 2023).

[35] Robinson DL . Accurate, low cost PM_2.5_ measurements demonstrate the large spatial variation in wood smoke pollution in regional Australia and improve modeling and estimates of health costs. Atmosphere 2020; 11: 856.

[36] Wang B , Chen H , Xenaki D , et al. Differential inflammatory and toxic effects in‐vitro of wood smoke and traffic‐related particulate matter from Sydney, Australia. Chemosphere 2021; 272: 129616. 33482518 10.1016/j.chemosphere.2021.129616

[37] Vardoulakis S , Giagloglou E , Steinle S , et al. Indoor exposure to selected air pollutants in the home environment: a systematic review. Int J Environ Res Public Health 2020; 17: 8972. 33276576 10.3390/ijerph17238972 PMC7729884

[38] Héroux ME , Anderson HR , Atkinson R , et al. Quantifying the health impacts of ambient air pollutants: recommendations of a WHO/Europe project. Int J Public Health 2015; 60: 619‐627. 26024815 10.1007/s00038-015-0690-y PMC4480843

[39] Weichenthal S , Pinault L , Christidis T , et al. How low can you go? Air pollution affects mortality at very low levels. Sci Adv 2022; 8: eabo3381. 36170354 10.1126/sciadv.abo3381 PMC9519036

[40] Marks GB . Misuse of pollution reference standards: no safe level of air pollution. Am J Respir Crit Care Med 2022; 205: 984‐985. 35275046 10.1164/rccm.202201-0160ED PMC9851491

[41] Borchers Arriagada N , Palmer AJ , Bowman DMJS , et al. Unprecedented smoke‐related health burden associated with the 2019–20 bushfires in eastern Australia. Med J Aust 2020; 213: 282‐283. https://www.mja.com.au/journal/2020/213/6/unprecedented‐smoke‐related‐health‐burden‐associated‐2019‐20‐bushfires‐eastern 32162689 10.5694/mja2.50545

