# Supplementary material for: Wood heater smoke and mortality in the Australian Capital Territory: a rapid health impact assessment
Source: Med J Aust. 2023 Nov 29;220(1):29–34. doi: 10.5694/mja2.52176 (PMC10952137; doi:10.5694/mja2.52176)
Supplement: Supplementary file 1 — Supplementary methods and results [file MJA2-220-29-s001.pdf]

Supporting Information

Supplementary methods and results

This appendix was part of the submitted manuscript and has been peer reviewed. It is posted as supplied by the authors.

Appendix to: Vardoulakis S, Johnston FH, Goodman N, et al. Wood heater smoke and mortality in the Australian Capital Territory: a rapid health impact assessment. *Med J Aust* 2024; doi: 10.5694/mja2.52176.

Figure 1. Monthly mean minimum temperature at Canberra Airport, by year*

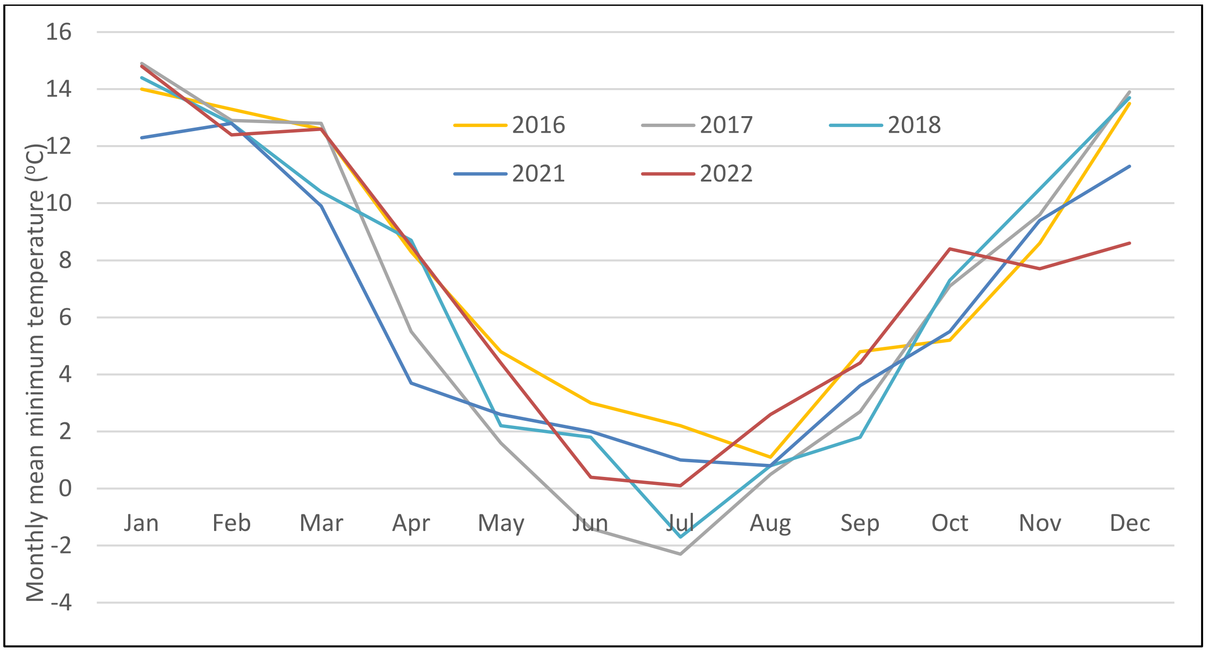

* Source: Australian Bureau of Meteorology. http://www.bom.gov.au/climate/data (viewed Sept 2023).

1. Diurnal patterns of $PM_{2.5}$ pollution

Figures 2 and 3 illustrate the diurnal pattern of wood heater pollution at the three ACT monitoring sites, showing how wintertime $PM_{2.5}$ pollution builds up in the evenings particularly at Monash and Florey, peaking after midnight. Emissions often increase when wood heater users turn down the air control for an overnight burn. A second smaller peak occurs in the morning when users reload or relight their heaters for the day. Lowest $PM_{2.5}$ levels are in the early afternoon when mean $PM_{2.5}$ during the wood heating season is lower than at other times of year.

Figure 2. Hourly mean $PM_{2.5}$ concentrations at (a) Monash, (b) Florey, (c) Civic by hour of day, season (wood heating = April to September, Rest-of-Year = January to March and October to December) and year (2015-2023). Means for Rest-of-Year are omitted for 2019 and 2020 because of the impact of the Black Summer Bushfires. Years 2015 and 2023 are presented for illustration only and are not included in the mortality impact analyses because of missing data. The last digit of each year (5-9, 0-3) distinguishes the year.

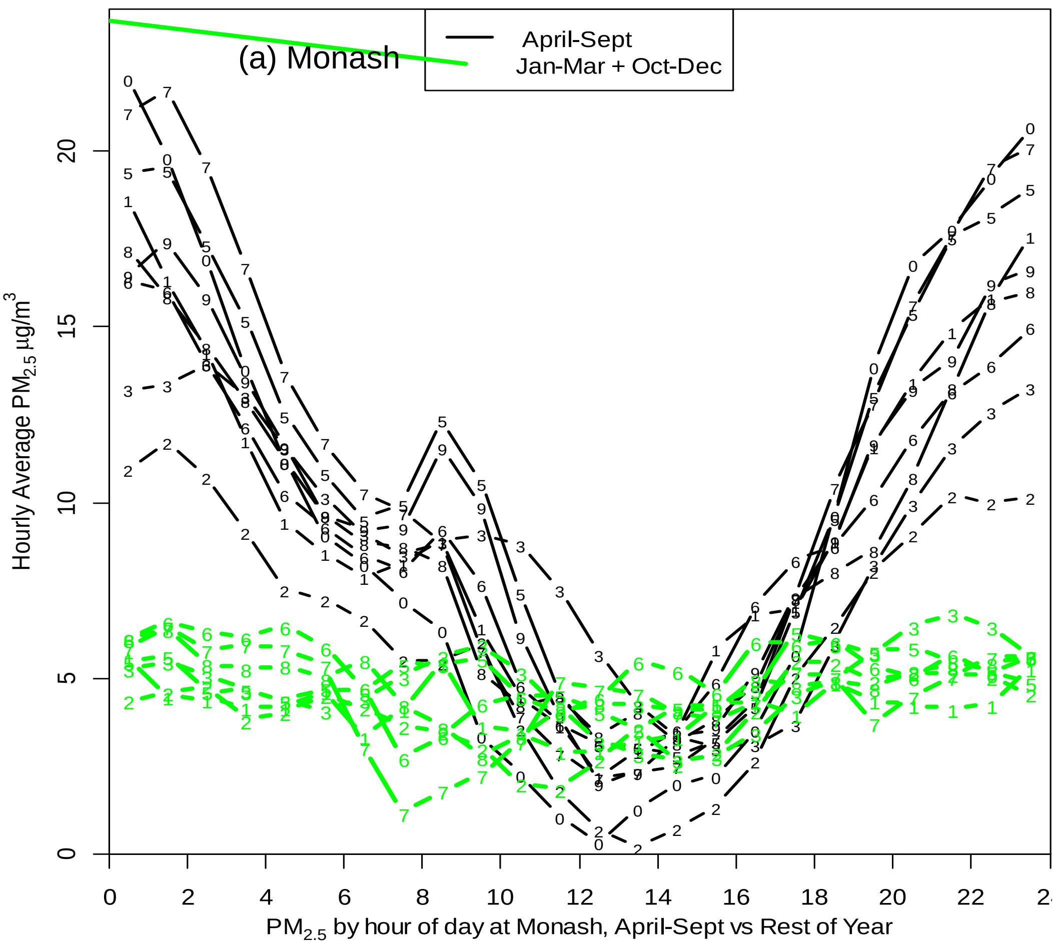

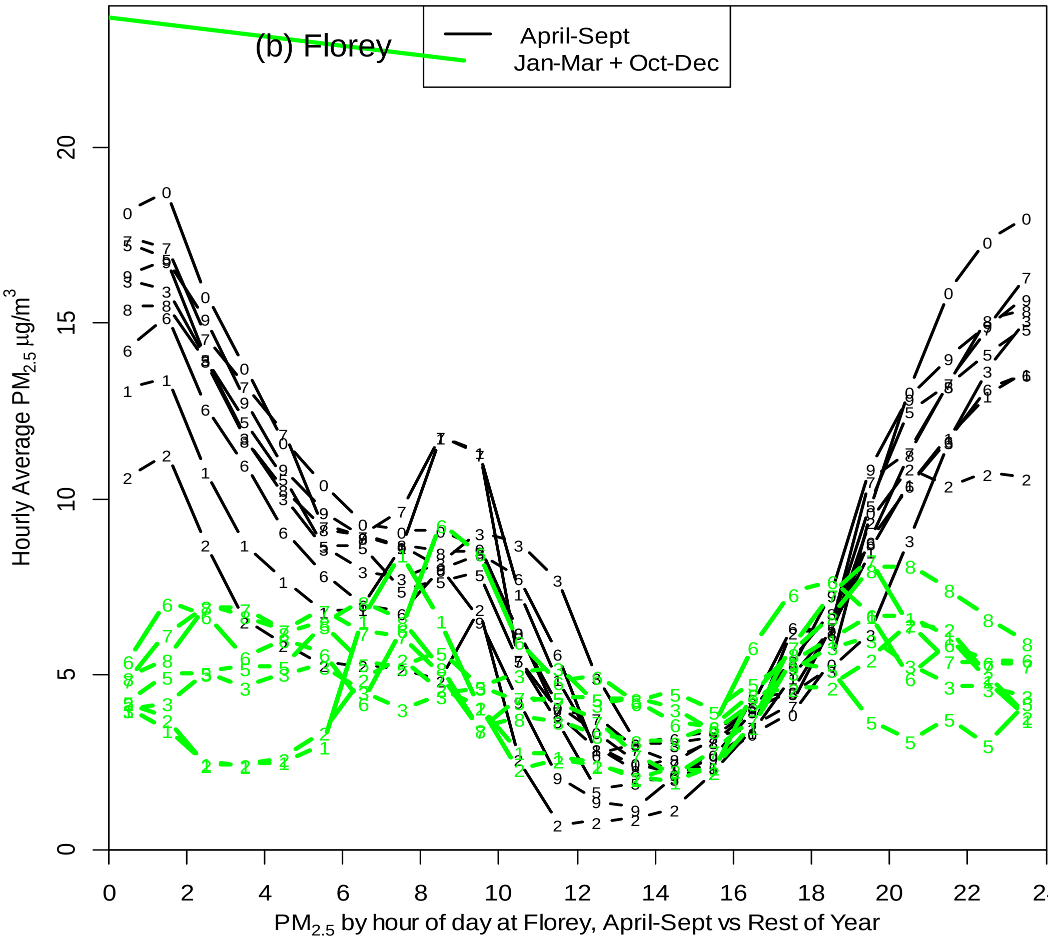
(b) Florey
April-Sept
Jan-Mar + Oct-Dec
Hourly Average $PM_{2.5}$ µg/m^3
PM2.5 by hour of day at Florey, April-Sept vs Rest of Year

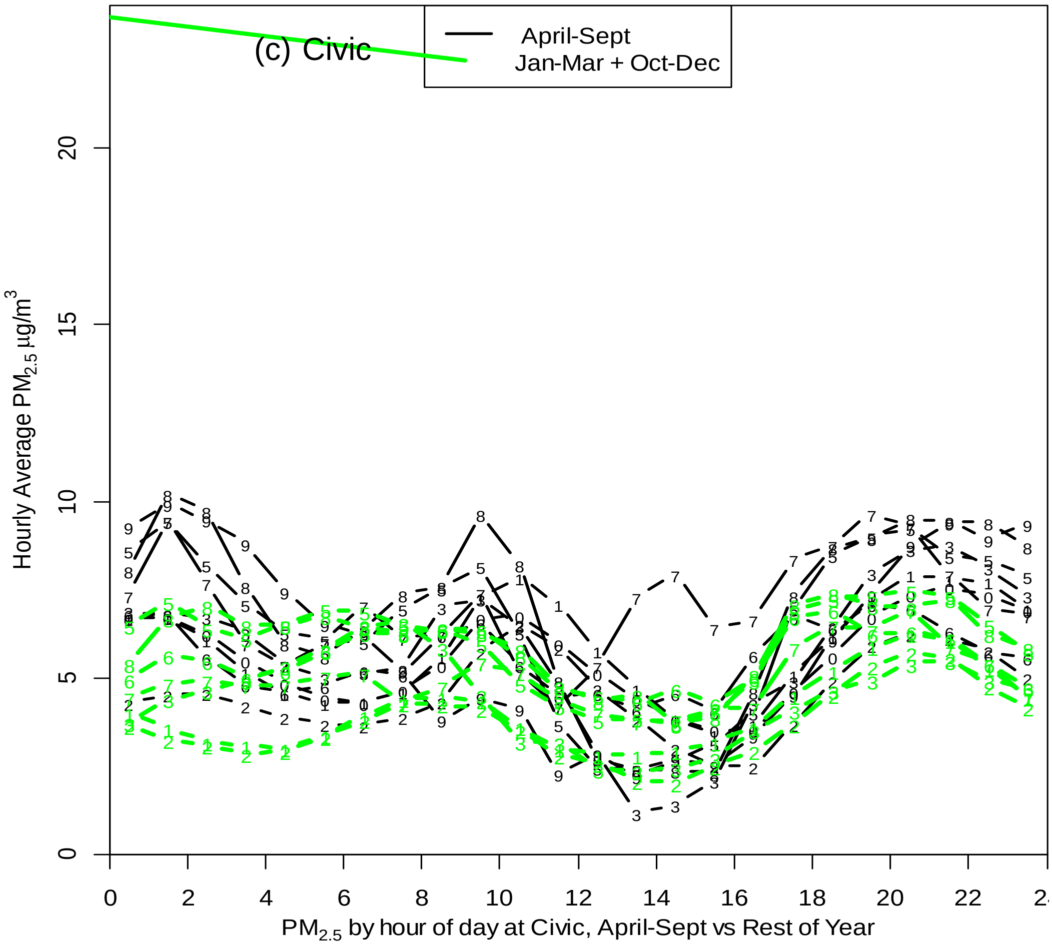
(c) Civic
April-Sept
Jan-Mar + Oct-Dec
Hourly Average $PM_{2.5}$ µg/m^3
PM2.5 by hour of day at Civic, April-Sept vs Rest of Year

Figure 3. Hourly mean $PM_{2.5}$ concentrations at (a) Monash, (b) Florey, (c) Civic by hour of day, the transition months (April, May, or September) and non-wood heating season (January-March and October-December). Means for the non-wood heating season are omitted for 2019 and 2020 because of the impact of the Black Summer Bushfires. Years 2015 and 2023 are presented for illustration only and are not included in the mortality impact analyses because of missing data. The last digit of each year (5-9, 0-3) distinguishes the year.

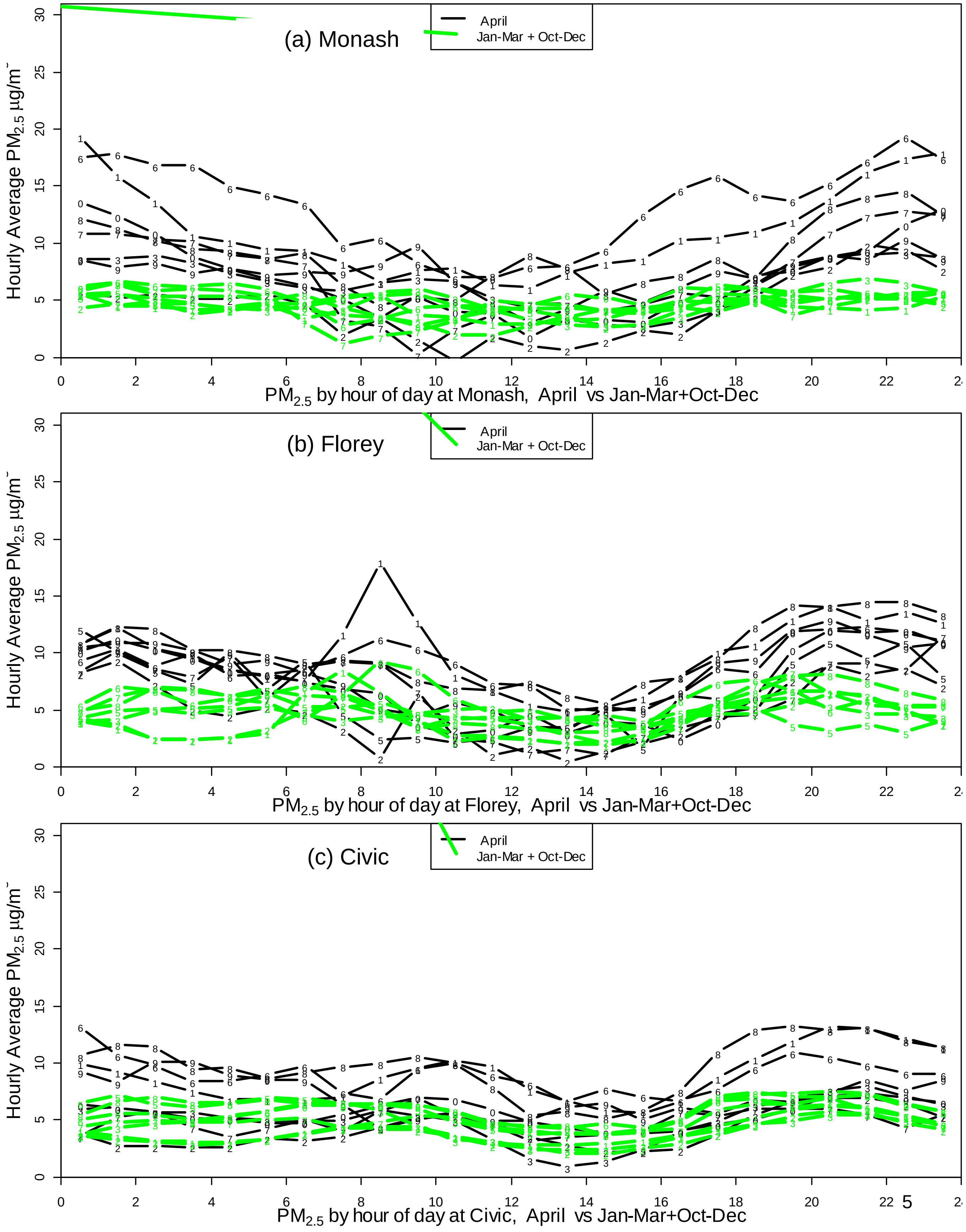

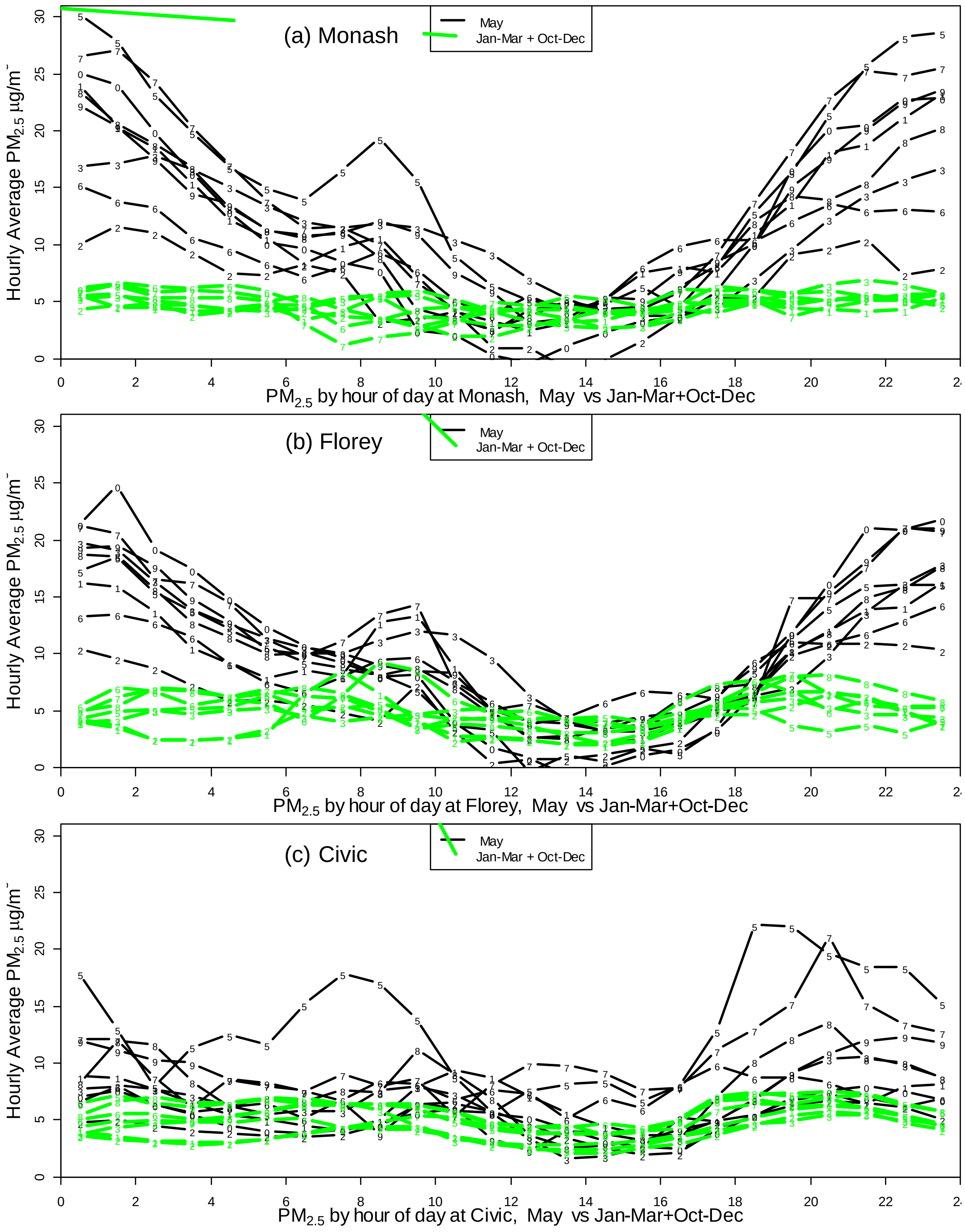
(a) Monash
May
Jan-Mar + Oct-Dec
Hourly Average PM2.5 µg/m
PM2.5 by hour of day at Monash, May vs Jan-Mar+Oct-Dec
(b) Florey
May
Jan-Mar + Oct-Dec
Hourly Average PM2.5 µg/m
PM2.5 by hour of day at Florey, May vs Jan-Mar+Oct-Dec
(c) Civic
May
Jan-Mar + Oct-Dec
Hourly Average PM2.5 µg/m
PM2.5 by hour of day at Civic, May vs Jan-Mar+Oct-Dec

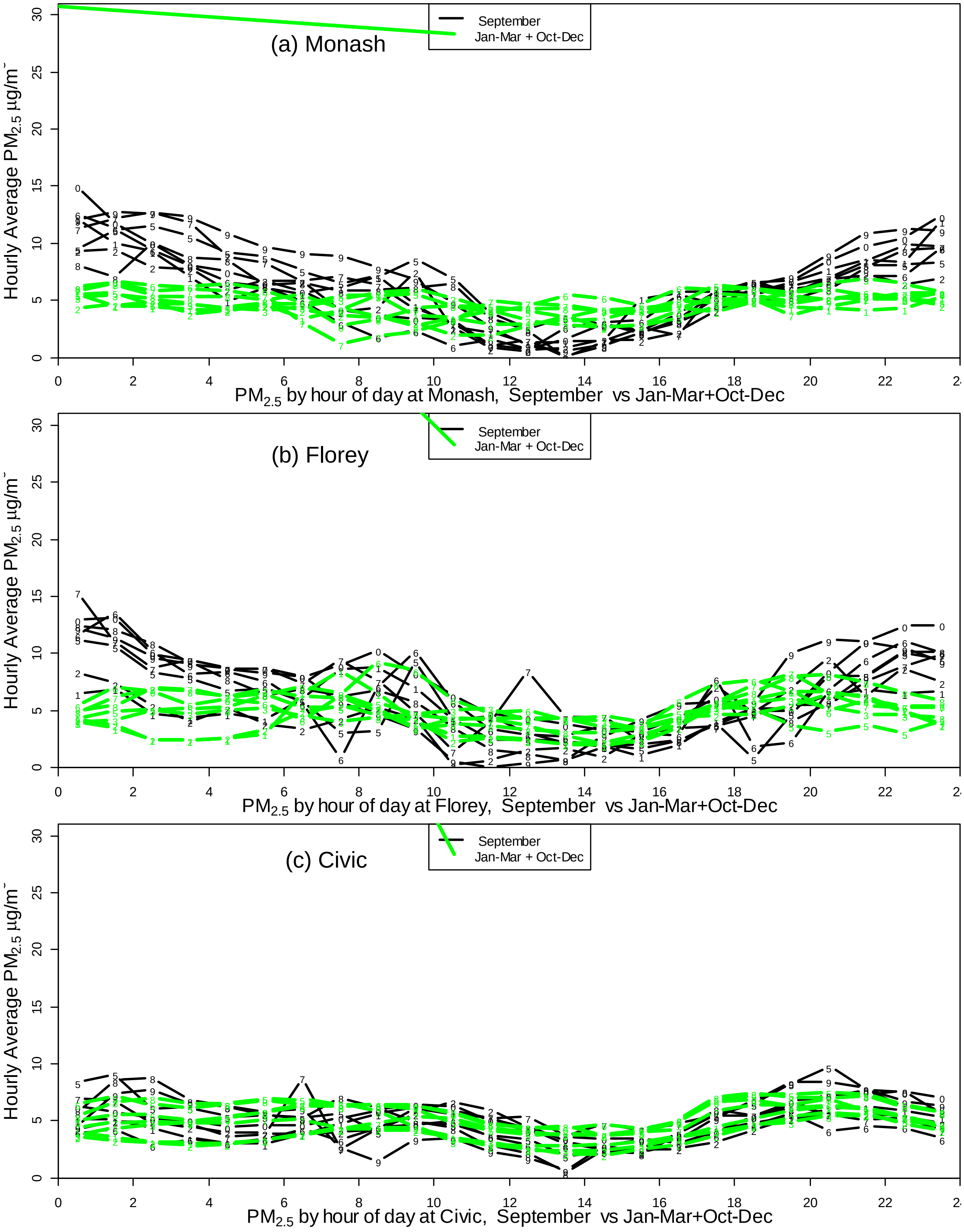

(a) Monash
September
Jan-Mar + Oct-Dec
Hourly Average $PM_{2.5}$ µg/m
$PM_{2.5}$ by hour of day at Monash, September vs Jan-Mar+Oct-Dec
(b) Florey
September
Jan-Mar + Oct-Dec
Hourly Average $PM_{2.5}$ µg/m
$PM_{2.5}$ by hour of day at Florey, September vs Jan-Mar+Oct-Dec
(c) Civic
September
Jan-Mar + Oct-Dec
Hourly Average $PM_{2.5}$ µg/m
$PM_{2.5}$ by hour of day at Civic, September vs Jan-Mar+Oct-Dec

2. Calculation of attributable mortality

As an example, we calculated deaths attributable to wood heater smoke in the ACT in 2021 by multiplying 90% of the 2160 total annual deaths (the 10% reduction accounts for deaths in early childhood, accidental deaths and suicides) with the attributable fraction (*AF*) of mortality associated with $PM_{2.5}$.

AF was calculated from the relative risk (RR_x) of mortality associated with long-term exposure to the wood heater $PM_{2.5}$ contribution of 1.49 μg/m^3 (Box 3) using the formula:

$$AF = (RR_x-1) / RR_x$$

The relative risk RR_x for a $PM_{2.5}$ increment of x = 1.49 μg/m^3 was calculated as:

$$RR_x = (RR_{10})^{x/10}$$

Where RR_{10} is the increase in natural cause mortality risk for 10 μg/m^3 increase in annual $PM_{2.5}$ exposure.

Using the RR_{10} = 1.209 derived from an Australian study[1] (Box 1), we estimated RR_x = 1.0287 and *AF* = 0.0279, resulting in 2160 × 0.90 × 0.0279 = 54.24 attributable deaths (Box 4).

Based on the Value of Statistical Life (VSL) of 5.3 million Australian dollars, the mortality costs of wood heater pollution in 2021 in ACT for RR_{10} = 1.209 was 54.24 × 5.3 = $287.5 million (Box 5).

Reference

1. Yu W, Guo Y, Shi L, Li S. The association between long-term exposure to low-level PM2.5 and mortality in the state of Queensland, Australia: A modelling study with the difference-in-differences approach. PLoS Med 2020; 17: e1003141.

Table 1. Mean $PM_{2.5}$ concentrations at Australian Capital Territory government monitoring stations, by year

	Mean $PM_{2.5}$ concentration (µg/m^3)				
**Monitor**	**Wood heater season (Apr–Sept)**	**Rest of year**	**Entire year**	**Wood heating proportion***	**Wood heating proportion****
Three-station mean					
2016	7.76	5.54	6.66	0.78 (12%)	1.00 (15%)
2017	9.27	4.95	7.11	1.51 (21%)	1.94 (27%)
2018	8.35	5.44	6.89	1.02 (15%)	1.31 (19%)
2021	7.86	4.16	6.02	1.30 (22%)	1.68 (28%)
2022	5.67	3.88	4.78	0.63 (13%)	0.81 (17%)
Monash					
2016	9.27	5.26	7.27	1.41 (19%)	1.81 (25%)
2017	11.08	4.53	7.84	2.32 (30%)	2.98 (38%)
2018	9.22	4.81	7.01	1.55 (22%)	1.99 (28%)
2021	9.64	4.18	6.95	1.94 (28%)	2.49 (36%)
2022	6.33	4.07	5.17	0.78 (15%)	1.00 (19%)
Florey					
2016	8.41	5.81	7.12	0.92 (13%)	1.18 (17%)
2017	9.67	5.34	7.50	1.51 (20%)	1.94 (26%)
2018	8.81	5.51	7.15	1.15 (16%)	1.48 (21%)
2021	8.07	4.26	6.17	1.34 (22%)	1.72 (28%)
2022	6.16	3.9	5.06	0.81 (16%)	1.04 (21%)
Civic					
2016	5.60	5.56	5.58	0.01 (< 1%)	0.02 (< 1%)
2017	7.05	4.98	5.99	0.71 (12%)	0.91 (15%)
2018	7.01	6.01	6.52	0.36 (5%)	0.46 (7%)
2021	5.86	4.04	4.95	0.64 (13%)	0.82 (17%)
2022	4.52	3.68	4.10	0.29 (7%)	0.38 (9%)

$PM_{2.5}$ = airborne particles of diameter smaller than 2.5 µm.
* Assumes that 70% of the difference between the annual mean concentration and mean concentration for periods outside the wood heater season is attributable to wood heater emissions.
** Assumes that 90% of the difference between the annual mean concentration and mean concentration for periods outside the wood heater season is attributable to wood heater emissions.

Table 2. Deaths attributable to wood heater pollution in the Australian Capital Territory, by year and exposure–risk function*

Relative risk source	Deaths (95% confidence interval)				
	2016	2017	2018	2021	2022
Chen & Hoek, 2020 (I)[1]	13 (10-14)	22 (17-25)	15 (11-17)	19 (15-22)	9 (7-10)
Chen & Hoek, 2020[1] (II)	26 (19-35)	46 (33-60)	31 (22-41)	39 (28-52)	19 (14-25)
Yu et al, 2020[2]	32 (23-43)	55 (39-75)	37 (27-51)	48 (34-65)	23 (16-31)

* Assumes that 70% of the difference between the annual mean concentration and mean concentration for periods outside the wood heater season is attributable to wood heater emissions.

Table 3. Deaths attributable to wood heater pollution in the Australian Capital Territory, by year and exposure–risk function**

Relative risk source	Deaths (95% confidence interval)				
	2016	2017	2018	2021	2022
Chen & Hoek, 2020 (I)[1]	17 (13-19)	29 (22-32)	19 (15-22)	25 (19-28)	12 (9-13)
Chen & Hoek, 2020 (II)[1]	34 (24-44)	58 (42-77)	40 (29-52)	50 (37-66)	24 (18-32)
Yu et al, 2020[2]	41 (29-56)	71 (50-96)	48 (34-65)	61 (44-83)	30 (21-40)

** Assumes that 90% of the difference between the annual mean concentration and mean concentration for periods outside the wood heater season is attributable to wood heater emissions.

Table 4. Equivalent cost of mortality attributable to wood heater pollution in the Australian Capital Territory, by year and exposure–risk function*

Relative risk source	Equivalent cost of deaths, $ million (95% confidence interval)				
	2016	2017	2018	2021	2022
Chen & Hoek, 2020 (I)[1]	69<br>(52-77)	119<br>(90-133)	80<br>(61-90)	103<br>(78-115)	50<br>(38-56)
Chen & Hoek, 2020 (II)[1]	139<br>(101-183)	242<br>(175-317)	163<br>(118-215)	209<br>(151-274)	101<br>(73-133)
Yu et al, 2020[2]	169<br>(120-230)	292<br>(208-397)	197<br>(141-269)	252<br>(180-343)	122<br>(87-166)

* Assumes that 70% of the difference between the annual mean concentration and mean concentration for periods outside the wood heater season is attributable to wood heater emissions.

Table 5. Equivalent cost of mortality attributable to wood heater pollution in the Australian Capital Territory, by year and exposure–risk function**

Relative risk source	Equivalent cost of deaths, $ million (95% confidence interval)				
	2016	2017	2018	2021	2022
Chen & Hoek, 2020 (I)[1]	88<br>(67-99)	153<br>(116-171)	103<br>(78-115)	132<br>(100-148)	64<br>(48-71)
Chen & Hoek, 2020 (II)[1]	179<br>(129-235)	310<br>(224-406)	209<br>(152-275)	267<br>(194-351)	130<br>(94-171)
Yu et al, 2020[2]	216<br>(154-294)	374<br>(267-507)	253<br>(180-344)	323<br>(231-439)	157<br>(112-213)

** Assumes that 90% of the difference between the annual mean concentration and mean concentration for periods outside wood heater season is attributable to wood heater emissions.

References

1. Chen J, Hoek G. Long-term exposure to PM and all-cause and cause-specific mortality: A systematic review and meta-analysis. Environ Int 2020; 143: 105974.
2. Yu W, Guo Y, Shi L, Li S. The association between long-term exposure to low-level PM2.5 and mortality in the state of Queensland, Australia: a modelling study with the difference-in-differences approach. PLoS Med 2020; 17: e1003141.
